# Getting to the heart of stem cell research: an interview with Christine Mummery

**DOI:** 10.1242/dmm.050270

**Published:** 2023-05-23

**Authors:** Christine Mummery

**Affiliations:** Department of Anatomy and Embryology, Leiden University Medical Centre, 2333 BE Leiden, The Netherlands

## Abstract

Professor Christine Mummery has pioneered the use of pluripotent stem cell models to investigate heart development and disease, and pushed the boundaries of what can be achieved with these versatile cells. In 2008, she became Chair of Developmental Biology at Leiden University Medical Centre, where she has refined and advanced *in vitro* models of the heart and now harnesses their clinical potential to screen drugs and personalise treatments for patients with various types of heart disease.

Christine has become integral to the stem cell community by promoting cross-disciplinary research and serving on several Ethical Councils, Scientific Advisory Boards and Editorial Boards. Her influence in the field of stem cell research led her to become the president of International Society for Stem Cell Research in 2020, and she has won numerous awards, including the Hans Bloemendal Medal 2014 for innovative interdisciplinary research, with Gordon Keller, the prestigious Lefoulon-Delalande Prize 2021 and the International Society for Stem Cell Research (ISSCR) Public Service Award 2023. In this interview, Christine shares her career trajectory, how disease modelling is shifting towards advanced *in vitro* systems and what challenges the field has yet to overcome.


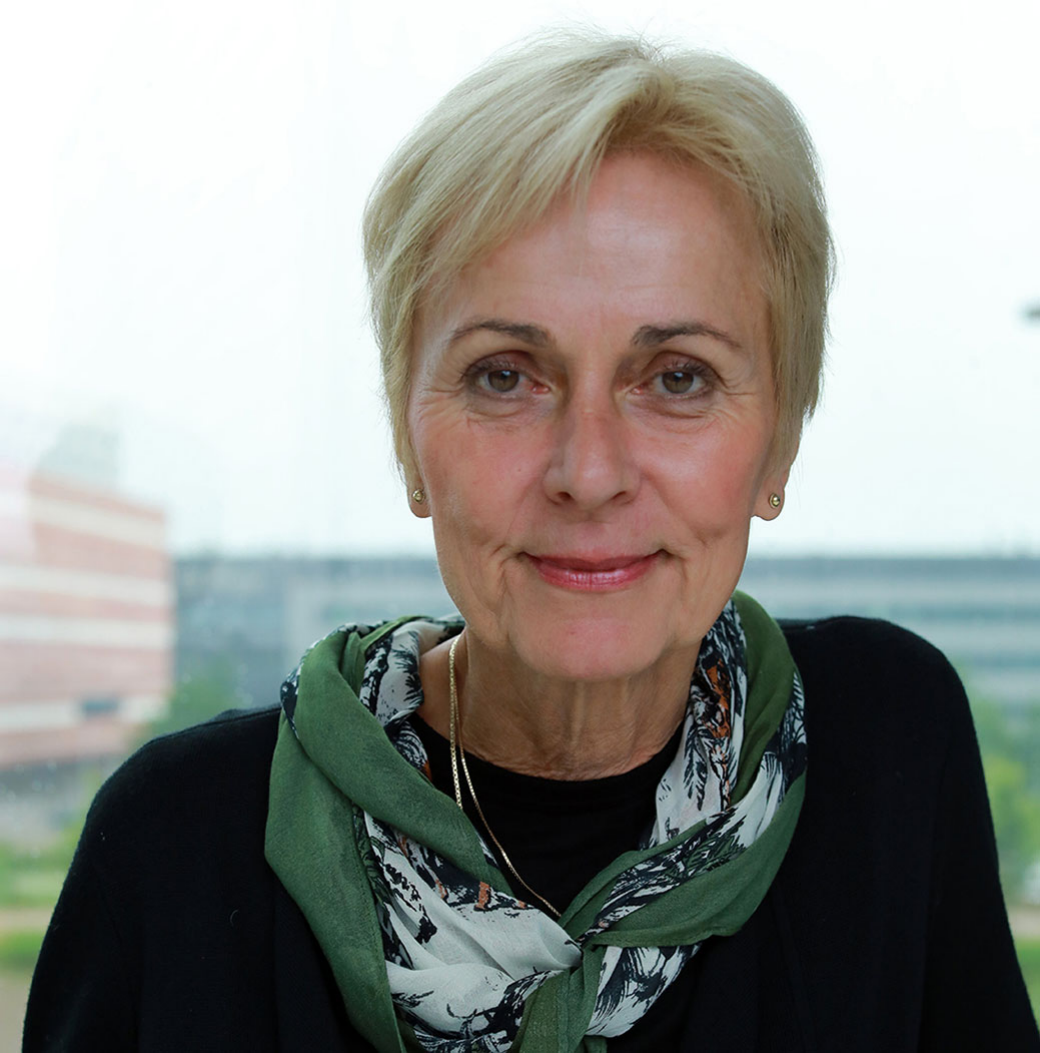


**After your undergraduate studies, what inspired you to switch to biophysics and how has this shaped your career?**

During my undergraduate studies in physics, I was always interested in what the cells were doing. For example, we learned about how they'd discovered DNA using crystallography, but we didn't learn very much about how DNA works, and I was kind of fascinated by that. I did a project on non-invasive imaging of the heart with ultrasound and became more and more fascinated by the medical application of physics in general. I then deliberately went to a university hospital – Guy's Hospital in London – to do a PhD, and I continued to work on ultrasound. I was hired to do the ultrasound part of the project, but I got very interested in the biology.

I then ended up applying for a postdoctoral position at the Hubrecht Institute in Utrecht. The lab was working on neuroblastoma, which is a brain tumour that forms from neuroblast nerve cell, and they were studying electrophysiology and ion transport in these cells. To begin with, I didn't know much about that area of biology at all, but I worked there for a couple of years as a postdoc, and I found it quite fascinating how cells from a tumour could differentiate. In those days, we thought differentiation could make them go from a malignant state to a benign state, so people were working on differentiation as a potential therapy.

It got even more interesting when I discovered there were things called pluripotent stem cells, which, at the time, were cells isolated from testis tumours in mice, known as teratocarcinoma stem cells. Then my group leader went to a meeting in Cold Spring Harbor and when he came back said, “I've just seen this fantastic thing, pluripotent stem cells, that are not tumorigenic; and they not only make neurons but make heart cells too”. I thought they were totally fascinating, so I went to Martin Evans’ lab in Cambridge to learn about these mouse embryonic stem cells (ESCs) and to Chris Graham's lab in Oxford to learn about human teratocarcinoma stem cells. Then I came back and established these techniques in our lab.

At the time, I was interested in development, and I started looking at how the mouse heart and vasculature develop using these mouse ESCs. I did that for a number of years until it became clear that human pluripotent stem cells, in the form of ESCs, were coming on the horizon. I had become friends with Alan Trounson and Martin Pera, who were then in Australia, and they offered their human ESCs (hESCs) to me. I went to Australia with two of my technicians, and we learned how to work with hESCs and brought that knowledge back to The Netherlands. I was then involved in helping draft the legislation to govern hESC derivation from surplus *in vitro* fertilisation (IVF) embryos in The Netherlands. In our very first experiment, the hESCs turned into beating heart cells. I was just amazed when they started beating, which was to me much more exciting than neurons that just sort of spread over the dish. If they had turned into neurons, I'd have probably gone back into neurobiology, but because they turned into heart cells, from then on, we worked on the heart. That's the way serendipity works.

“I was just amazed when they started beating, which was to me much more exciting than neurons that just sort of spread over the dish. If they had turned into neurons, I'd have probably gone back into neurobiology, but because they turned into heart cells, from then on, we worked on the heart. That's the way serendipity works.”

I spent quite a lot of time using cardiomyocytes from hESCs as heart transplants in mice with myocardial infarction. We got quite nice grafts in the mice, and we could make the heart function better, but they were young mice and we treated them immediately after a myocardial infarction. Once I saw the heart that had been taken out of a patient who had had a myocardial infarction, and I saw this organ full of fibrosis that looked awful and full of fat, I thought, “How on earth, with even billions of beating cells, are we ever going to fix this?”. So, I decided that research area wasn't the way to go for me and I didn't feel I would be up to that challenge. I always say to somebody like Chuck Murry, who has carried on, “I think you're really heroic that you battled on with that”. When I see that, 10-12 years later, they're making progress but still battling on, I think that our energies were better spent making disease models and trying to understand the cells.

I then went to the Harvard Stem Cell Institute to answer a specific question: if we change the shape of the ESC-derived heart cells, would we make them mature? When the ESC-derived heart cells were cultured, they were immature and had a triangular shape, but when they're in the adult body, they're oblongs. I went to Kit Parker's lab, where they printed a matrix as little oblong shapes onto a plastic plate, and we put the cells on top. In doing this, we saw some structural maturation, but not a lot of electrical maturation, in the heart cells.

The Harvard Stem Cell Institute was set up as a private institute within Harvard to circumvent the problem with the National Institutes of Health (NIH) funding human embryonic stem cell research. Doug Melton derived many hESC lines, to protect the cell lines against the government regulations. However, while I was at the Harvard Stem Cell Institute, Shinya Yamanaka published his paper on induced pluripotent stem cells (iPSCs) (Takahashi and Yamanaka, 2006). It was a very thrilling time to be there and to see the dilemma in Harvard on whether to continue research with hESCs when there were these human iPSCs (hiPSCs). At the institute, they did a remarkable switch, and in some ways became leaders in hiPSCs for a certain time. George Daley was among the first to make hiPSCs there and he generously gave me the constructs to make hiPSCs at the Hubrecht Institute when I returned in 2008.

Then, we repeated what we'd done with hESCs with hiPSCs, and we found we could also make them form cardiomyocytes, and the road was set. We then asked if we could derive them from patients, could they model disease and if we could make them more mature. Now, we are still making disease models with iPSCs, still hoping for personalised medicine and still trying to solve how best to induce their maturation.

**Could you outline the main advantages for using stem cell-derived *in vitro* systems to study the heart and heart-related disease?**

The bottom line is that the mouse heart beats very fast – about 500 times a minute. Of course, that's not what a human heart does, as it beats about 60 times a minute. So, it struck me, as a physicist, that there must be something very different between the two, and it should lie in the ion channels. And, in fact, that is where the difference lies, and the electrophysiology of this fascinated me.

When we got beating cells from hESCs or hiPSCs, even though they were immature, the full complement of ion channels was present. This meant there were many diseases we could model, such as channelopathies, which are caused by mutations in cardiac ion channels. If you look at the literature, you'll see an incredible number of papers modelling ion channel diseases and looking for treatments. That's because even in the very simplest systems, you've got the ion channels.

One of the most important things we knew about drugs going into the clinic was that many cause serious side effects, and an unacceptable number cause sudden cardiac death. In humans, if we go from 60 to 180 or 240 beats per minute, we're very ill or even dead, and this is what was happening in response to some drugs going into the market. That meant that the mice being used to test the drugs weren't revealing the risk to humans. After a lot of work, it turned out that hESC- or hiPSC-derived cardiomyocytes were better predictors of risk than the mouse, but also better than rabbits, which was the model you were supposed to use if you were going to assess cardiotoxicity. That's why the U.S. Food and Drug Administration (FDA) are now making these models part of their safety dossiers (Pang et al., 2019). We contributed to making that a success, and I think it won't be very long before experiments assessing cardiotoxicity will be entirely on human cells, because they're more predictive.

“We contributed to making that a success, and I think it won't be very long before experiments assessing cardiotoxicity will be entirely on human cells, because they're more predictive.”

We also wanted to use these stem cell models to develop new drugs that don't cause side effects like heart failure. To do this we developed more mature iPSC models that allow us to study heart failure. As an example: the only drug for treating children with the bone tumour osteosarcoma is doxorubicin, which causes 10% of them to get heart failure, and the only treatment for them is a heart transplant, which may not last into adulthood. There are, in fact, ethical issues about whether you should treat a child with osteosarcoma with those drugs. We're working with some great medicinal chemists who develop variants of doxorubicin that don't have toxic side effects on the heart, but still kill the tumour (Qiao et al., 2020). We test their candidates in our *in vitro* system and now we have candidates that are moving to the clinic.

It's likely that hiPSCs are also more predictive for drugs that will benefit patients. In a best-case scenario, about one in four patients will respond to prescribed drugs; in the worst case, even for commonly prescribed drugs, it's only one in 20. We believe that stem cell models can provide a huge save in healthcare system costs by predicting who will respond. For instance, doctors test breast cancer patients to see whether they're likely to benefit from chemotherapy, and patients who won't benefit can avoid having chemotherapy. We think it will be possible, in the future, to do similar things with many drugs: don't give them to a patient if they end up only having the side effects, but no benefit. There's lots of exciting things to do, even though it might not be that we're going to transplant the cells into patients.

**What discovery or research project have you found most exciting during your career?**

A recent exciting project was with Milena Bellin and Valeria Orlova. We found a way of making cardiomyocytes more mature by mixing them with cardiac stromal cells and endothelial cells (Giacomelli et al., 2020). For us, that was game changing, because, in a sense, we understood suddenly why you need cells in 3D and why they need their specific environments, and we also discovered something about the mechanism of cardiomyocyte maturation. We're now exploring this mechanism much more deeply to see whether we can harness it.

Ultimately, using this co-culturing technique, we can mature hiPSC-derived cardiomyocytes to a postnatal stage. And we know that they are at a postnatal stage because Milena has recently found a splice variant of a particular gene that is only expressed when the cardiomyocytes are mature – in babies after they're born, and later (Campostrini et al., 2022). So, if we detect this splice variant, we know we've actually passed this critical birth borderline. I think that's very cool. We can study diseases where, for example, a splice variant is only expressed postnatally. We can test drugs that might only have an effect after birth, not on a foetus. So, I think that's probably one of our most recent exciting breakthroughs.

**What have been the most astounding recent developments in stem cell research?**

I think the sudden growth of disease modelling, in general, has been very exciting. I think one of the illustrations of this is that Roche, the pharmaceutical company, is investing €2.5 billion in drug research that includes using these *in vitro* disease models. They're using mostly organoids of the type Hans Clevers uses, but they're also using iPSCs. Second, I think tissue engineering and organ-on-chip are up and coming. Roche has also given €400 million to set up a new organ-on-chip institute in Basel. That will be headed by Matthias Lütolf, who's an engineer but a sort of transformed engineer in the same way that I'm a transformed physicist. He's going to use organ-on-chip for disease modelling and drug discovery. There is a lot of interest in developing these models, because they're not just cells, they've got microfluidics in them, which means that you can mimic blood flow, lymph vessel flow or even air flow, if it's a lung model. They still have to meet their promise. I don't think they've actually delivered much more than what we have in some stem cell models yet, but I think they will deliver interesting things.

We certainly see some effects in 3D models that we don't see in 2D, which I think is encouraging. The closer we get to real tissue; it seems the better the tissue response. It was one of the reasons we set up the Netherlands human disease modelling technology organisation [Human Organ and Disease Model Technologies (hDMT)] in 2015. We linked up the engineering with the biology to form this organisation and managed to use that as a springboard to get a €20 million grant to work on organs-on-chip further, which led to European Union (EU) grants. Then, we established the European Organ-on-Chip Society, because there were about five or six small workshops and meetings a year, so we organised one annual meeting that all the organ-on-chip people in Europe usually go to.

**Do you think there is a movement to use cells that are from more diverse populations?**

Yes, indeed. Very often, the ethnic background of patients is not noted when cells are collected from them. So, there are some efforts now to do this systematically, as I think there are only two iPSC lines derived from African people. Of course, genetic disease of the heart can affect different populations differently, so it's very important to use cells from different genetic backgrounds.

If you want to use cells from lots of patients, and do a population study, you're going to need robotics, as you can't do this by hand. We're now developing robotics to reprogramme cells, make them grow, freeze them, differentiate them and do the analysis. Some parts of this process we've already got the robotics doing, allowing us to do high-throughput analysis of our cardiac microtissues' response to drugs. This is, again, where engineering has got to come into our research, and we're going to need companies who develop the robotics in robust ways.

**You co-founded the spin out Ncardia. How do you decide when a research project is mature enough for this type of progression?**

It was actually one of my PhD students who, from the beginning, was super keen to form a spin out. He was the first to test cardiotoxicity in our model systems and then made a pitch to an academic organisation that supports start-ups and got about €0.5 million. We set up what was originally called Pluriomics in 2012, and later become Ncardia. The only advice I gave him was to make sure he had a product at the outset, as it might be a safer bet if he had got something to sell. They refined our methods for making cardiomyocytes, which was extremely good – they're beautiful cardiomyocytes. In fact, their protocol is so good that it's the one BlueRock adopted for their clinical trials. So, he sold cardiomyocytes and endothelial cells, and we patented the endothelial differentiation method. They've made a huge success of Ncardia, and now have a large investment to take iPSC derivatives into the clinic. They've been much more successful than if I'd have been running the company. I think it is a success story, despite me, rather than thanks to me, I suppose.

We still have young researchers in our department that are really smart and keen to have start-ups. I would support them all the way, even though I wouldn't necessarily do it myself. The interesting thing is that even really top scientists in the regenerative medicine field are moving over to companies. These excellent academics are getting frustrated by how slow things move to the clinic from academia, and I think they're quite right. We will always be making models, but the moment that they're any good, we will be passing them on to biotech or pharma because research doesn't really become applied unless you involve commercial entities.

We got a new grant from Novo Nordisk called reNEW, which is very interesting because about half of it is for basic research, and the other half is for translation to industry and eventually the clinic. I don't think there are many grants that actually visualise academics taking something through to commercial success. We have business developers watching the work we're doing and helping us commercialise it. I think it's very forward thinking of Novo Nordisk to invest such a lot of money – €300 million – for 10 years over three sites (Copenhagen, Melbourne and Leiden) on a project like this.

**Could you outline how regulations/guidelines in bioethics and stem cell research are continuing to change?**

We went through a phase in which hiPSCs were welcomed as the ethically free counterpart of hESCs. But now we're going up a steep ethical slope again, regarding informed consent, transparency, commercialisation and whole-genome sequencing privacy. For instance, ethical concern is arising around waste material from surgery. It used to be an anonymised source, and we could just have access to it. Now with genome identity technology that exists, we could deanonymise it without intending to. We could discover things about patients, or even healthy individuals, that they wouldn't want to know.

“We went through a phase in which hiPSCs were welcomed as the ethically free counterpart of hESCs. But now we're going up a steep ethical slope again, regarding informed consent, transparency, commercialisation and whole-genome sequencing privacy.”

Furthermore, some of our older cell lines don't have proper consent paperwork, according to present-day regulations, so we have to do reconsents. In some cases, this means we might have to collect new donor samples and start again. There are also some philosophers and sociologists who think patients should have a longer period to change their mind. Which, in some ways, I appreciate, because if you donate when you're 25 years old, you may change your mind when you're 55. It's even more apparent if you generate a cell line from umbilical cord blood. The guardians or parents would have given permission, so should you reconsent when the child turns 18 or not? These are difficult questions, and it can make investments in these cell lines very high risk. Just imagine if you've spent €2 million and made a load of cells to treat patients, and then the patient or healthy donor changes their mind. Who's going to cover those costs? It would also be extremely difficult tracing the cell lines to all the different labs they have been sent to and asking people to destroy them and all of their modified derivatives.

Most guidelines currently say that you can withdraw consent until the cell lines start being made, but, after that, once those investments have been made, you can't. The ISSCR is making a new set of guidelines right now on how you can best manage this because it could make the field very difficult.

**What do you enjoy doing outside of the lab and work?**

We have a sailing yacht and this summer we went to Sweden and Denmark on the boat. I can even do Zoom calls from the boat, so I went away for 5 weeks, and my husband went for 7 weeks. It was really nice. The year before we went down the coast of France and before we've gone across to the UK. I can't say I'm a true sailor, but I do what the captain says. And we take the dog, so it's lovely.

## Supplementary Material

10.1242/dmm.050270_sup1 Supplementary information
