# Supplementary figures and images for: Getting to the heart of stem cell research: an interview with Christine Mummery
Source: Dis Model Mech. 2023 May 23;16(5):dmm050270. doi: 10.1242/dmm.050270 (PMC10233713; doi:10.1242/dmm.050270)

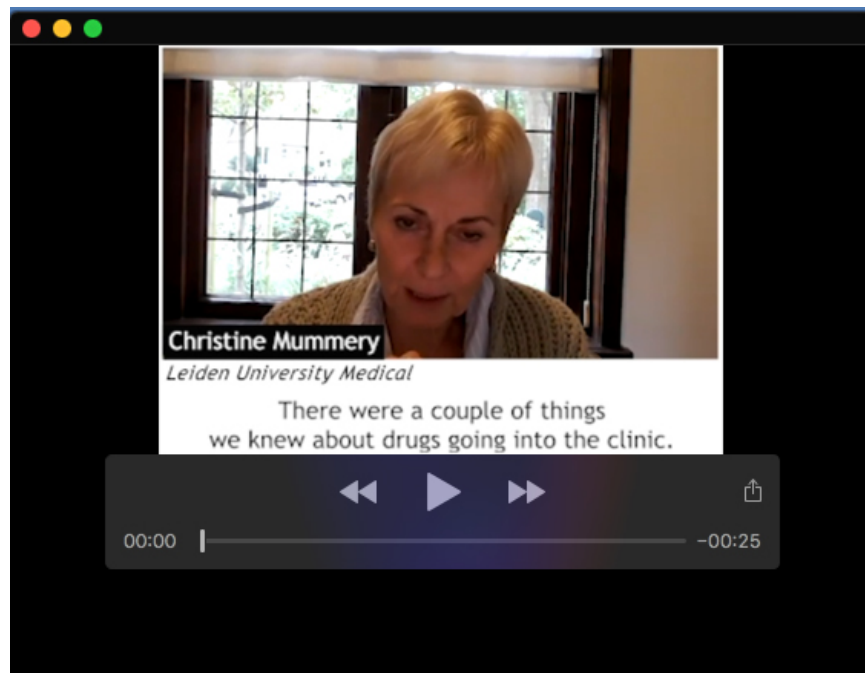

**Movie 1.**

Supplement: Supplementary information [file dmm-16-050270-s1.pdf]
